# Nonlinear correlation and mediation effects between serum 25-hydroxyvitamin D levels and all-cause mortality in COPD patients

**DOI:** 10.3389/fnut.2024.1412606

**Published:** 2024-06-04

**Authors:** Qi Jiang, Yuewen Jiang, Zheru Ma, Jingda Huang, Yang Li

**Affiliations:** ^1^Department of Respiratory and Critical Care Medicine, First Hospital of Jilin University, Changchun, China; ^2^Department of Respiratory and Critical Care Medicine, Qiyang People's Hospital, Yongzhou, China; ^3^Orthopaedic Center, First Hospital of Jilin University, Changchun, China; ^4^Department of Nephrology, The First Hospital of Jilin University, Changchun, China

**Keywords:** COPD, mortality, vitamin D, cohort study, NHANES

## Abstract

**Background:**

Numerous studies have shown that low levels of vitamin D are linked to a higher risk of inflammatory diseases and their progression. However, how vitamin D levels affect mortality in chronic obstructive pulmonary disease (COPD) patients is still unclear. Thus, this study aimed to explore the relationship between serum 25-hydroxyvitamin D [25(OH)D] levels and the risk of death from all causes in U.S. adults with COPD.

**Methods:**

This study analyzed 1,876 adults with COPD from the National Health and Nutrition Examination Survey (2005–2018). Mortality data up to December 31, 2019, were obtained from the National Death Index (NDI) records. Participants were categorized into three groups according to their 25(OH)D levels: Q1 (<50.0 nmol/L) for deficiency; Q2 (50.0–74.9 nmol/L) for insufficiency; and Q3 (≥75.0 nmol/L) for adequacy. A weighted Cox regression model assessed the link between 25(OH)D levels and mortality. Kaplan–Meier survival curves, subgroup, and sensitivity analyses were conducted. Additionally, the relationship between 25(OH)D and the hazard ratio (HR) was detailed through restricted cubic spline analysis. Mediation analysis revealed how 25(OH)D mediates the relationship between Dietary Inflammatory Index and mortality.

**Results:**

There were 395 all-cause deaths during the follow-up, resulting in a mortality rate of 21.06%. After adjusting for potential confounders, higher 25(OH)D levels significantly correlated with a lower risk of all-cause mortality in COPD patients (HR = 0.52, 95% CI: 0.37–0.72, *p* < 0.001). Restricted cubic spline analysis indicated a non-linear relationship between 25(OH)D levels and all-cause mortality (*p* for nonlinear = 0.023), with levels below 63.4 nmol/L posing an independent risk for all-cause mortality in COPD patients (HR = 0.98, 95% CI: 0.97–0.99, *p* = 0.005). Sensitivity and subgroup analyses confirmed our results’ robustness, with mediation analysis showing 25(OH)D’s 22% mediating effect on diet-induced inflammation and all-cause mortality in COPD patients.

**Conclusion:**

25(OH)D independently lowers the risk of all-cause mortality in COPD patients, with a non-linear L-shaped correlation, and mediates the effect of Dietary Inflammatory Index on mortality, suggesting new therapeutic possibilities.

## Background

Chronic obstructive pulmonary disease (COPD) has the highest mortality rate among chronic respiratory diseases (1). According to the World Health Organization, COPD is projected to become the third leading cause of death globally and the fourth leading cause of death in the United States in the next decade. The disease burden caused by COPD is expected to slowly and steadily increase (2, 3). Characterized by persistent airflow limitation and chronic inflammation of the airways, COPD ranks among the leading causes of mortality and morbidity, resulting in a substantial socioeconomic burden (4). To date, the pathological mechanisms of COPD are attributed to excessive inflammation, dysfunctional oxidative stress, and imbalance of protease-antiprotease systems (5–7).

As a type of steroid hormone, vitamin D can have unique biological effects on many target organs. It is most well-known for its role in bone calcium metabolism and maintaining the homeostasis of bone and calcium (8). Additionally, many studies also have revealed the unique effects of 25-hydroxyvitamin D on various cellular processes, such as cell proliferation, differentiation, wound healing, repair, and involvement in the host immune and inflammatory regulatory systems (9). The deficiency of 25-hydroxyvitamin D has been confirmed to be associated with the progression of multiple COPD pathogenesis processes, including inflammation regulation, excessive oxidative stress, increased protease expression, impaired host defense, and pulmonary airway remodeling (10). Evidence from clinical trials and meta-analyses indicates that 25-hydroxyvitamin D supplementation plays a role in reducing COPD exacerbations and improving disease prognosis (11–14).

Previous studies have highlighted vitamin D’s potential to reduce mortality rates across diseases and its role in delaying COPD progression (15, 16). However, there is limited current scientific research on the association between vitamin D and mortality rates in COPD patients (17, 18). Specifically, no comprehensive study has explored the direct effect of vitamin D on mortality risk in COPD patients among the non-institutionalized population in the United States. This gap is critical for understanding vitamin D’s role in COPD management because the non-institutionalized population represents a wider range of COPD patients, making the research findings more applicable and practical. This study will analyze NHANES data from 2005 to 2018 to investigate the relationship between serum 25-hydroxyvitamin D levels and all-cause mortality in COPD patients, as well as explore the potential impact of varying levels of serum 25-hydroxyvitamin D on mortality in COPD patients. The NHANES database, which contains nationally representative samples, offers a unique opportunity to address this research gap. The database covers a diverse population and ensures data accuracy and quality through standardized data collection processes. This solidifies the foundation for conducting reliable and effective statistical analyses. This study aims to fill gaps in the existing literature and enhance understanding of vitamin D’s potential role in reducing mortality risk among COPD patients.

## Methods

### Study design and data source

This study analyses data from the US National Health and Nutrition Examination Survey (NHANES) database, focusing on the years 2005–2018. The primary objective is to explore associations between serum 25-hydroxyvitamin D levels and long-term mortality rates in community-dwelling adults with COPD. Additionally, the study examines the potential mediating role of serum vitamin D in the relationship between the dietary inflammation index and mortality.

The NHANES database comprises data collected by the National Center for Health Statistics (NCHS), part of the CDC in the United States. The survey is designed to assess the health and nutritional status of individuals from various age groups nationwide. A sophisticated, multistage design in survey procedures ensures the data’s representativeness of the US population, excluding those in institutional settings. Researchers are granted access to use the data for research, made available by the NCHS.

NHANES survey participants first undergo a household interview and are then invited for a comprehensive examination at a mobile examination center (MEC). The examination encompasses physical measurements, specialized tests, and lab assessments. Consequently, participant evaluations from the NHANES database are deemed reliable and comprehensive, similar to population-level assessments (19). More information on the NHANES survey is available at its official website: https://www.cdc.gov/nchs/nhanes/index.htm. Note that all participants provided written informed consent for the NHANES survey.

### Study population selection

**Figure 1:**
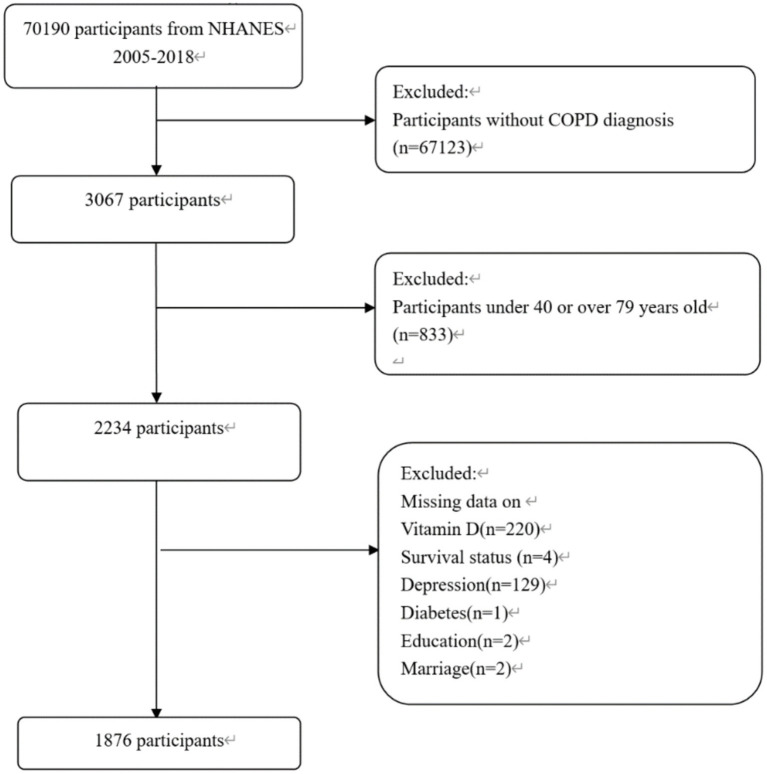
Cohort flow diagram in NHANES 2005–2018.

The study included community-dwelling adults aged 40–79, diagnosed with COPD by a physician between 2005 and 2018 in the NHANES dataset. COPD identification from the NHANES questionnaire was based on affirmative responses to questions about a doctor’s diagnosis of COPD, chronic bronchitis, or emphysema. This method for identifying COPD patients has been used effectively in many previous studies using NHANES data (20, 21). Participants who answered “yes” to any of these questions were considered COPD patients. From the initial 70,190 participants, 1,876 were included in the study after excluding those without vitamin D levels, survival data, or covariates, and those who could not be diagnosed with COPD. The sample is representative of 11,221,247 individuals in the United States, with the screening process detailed in Figure 1.

### Mortality status

Since late 2019, NHANES participants have been linked to the National Death Index (NDI) database, which contains nine cause-specific death categories. This link facilitates the identification of mortality patterns and primary causes of death. Detailed information on mortality files and cause-specific definitions can be found at the CDC Data Link – Mortality Public Information.

### 25-Hydroxyvitamin D measurements

The serum concentration of 25-hydroxyvitamin D (25(OH)D) serves as the biomarker for assessing vitamin D status. Serum 25(OH)D level classifications follow guidelines from the Endocrine Society Clinical Practice (22). The classifications are as follows: Q1 (<50.0 nmol/L) for deficiency; Q2 (50.0–74.9 nmol/L) for insufficiency; and Q3 (≥75.0 nmol/L) for adequacy.

### Covariates

Demographic data collected included age (40–49, 50–59, 60–69, 70–79), gender (male/female), race/ethnicity (non-Hispanic black, non-Hispanic white, Mexican American, other), education level (less than high school, high school equivalent, higher), marital status (married/partner, widowed/divorced/separated, single), and smoking status (never, former, current). “Never smoked” refers to individuals with less than 100 cigarettes in their lifetime, “Former smokers” to those who quit smoking after the same threshold, and “Current smokers” to those still smoking after 100 cigarettes.

Hypertension was identified if participants reported a diagnosis on multiple visits, received prescription recommendations, or had mean systolic ≥140 mm Hg or diastolic ≥90 mm Hg across three measurements. Diabetes mellitus (DM) was confirmed by positive responses to insulin use, physician-diagnosed diabetes, or blood sugar control medication. A CVD history was determined by positive responses to doctor-diagnosed myocardial infarction, angina, coronary heart disease, or stroke. The MetS group included individuals meeting at least three criteria: (1) triglycerides >150 mg/dL; (2) waist circumference ≥ 102 cm for men or ≥ 88 cm for women; (3) HDL levels ≥40 mg/dL for men or ≥ 50 mg/dL for women; (4) blood pressure ≥ 130/≥85 mm Hg; and (5) fasting blood glucose ≥110 mg/dL. Depressive status was assessed using participants’ PHQ-9 questionnaire responses. The PHQ-9 is a 9-item self-report depression scale assessing symptom frequency over the past 2 weeks. Items are scored from 0 (none) to 3 (almost daily). PHQ-9 scores range from 0 to 27, categorized into two groups: “not depressed” for scores <5 and “depressed” for scores of 5 or higher. The DII was calculated based on the 24 h dietary recall data from day one. DII calculation incorporated 26 dietary parameters, including carbohydrates, protein, total fat, saturated fat, PUFA, n-3 fatty acids, cholesterol, energy, alcohol, fiber, folate, iron, magnesium, zinc, selenium, MUFA, caffeine, niacin, riboflavin, thiamine, beta-carotene, and vitamins A, B6, B12, C, and E. Initially, calculate the subjects’ average nutrient intake, subtract the global mean, and divide by the standard deviation to obtain Z-scores. Next, Z-scores are converted to percentiles, doubled, and reduced by one to recenter the data. Multiply the central percentile value of each parameter by its inflammatory effect score to calculate a “food-specific DII score.” Lastly, values are combined for an “overall DII score” (23–25).

### Statistical analysis

To mitigate bias from oversampling, we applied sample weights as per NHANES guidelines. We present normally distributed continuous variables with mean and standard deviation, and categorical variables with frequency and proportion. We used ANOVA for continuous variables and Pearson’s chi-square for categorical variables to assess mean differences and proportions. Serum 25-hydroxyvitamin D concentration was treated as a categorical variable. The Kaplan–Meier model assessed cumulative all-cause mortality rates among COPD participants with varying serum 25-hydroxyvitamin D levels. We utilized the Cox proportional hazards model to examine the effect of varying 25-hydroxyvitamin D levels on all-cause mortality in COPD patients. Model 1 featured a univariate analysis of 25-hydroxyvitamin D levels; Model 2 adjusted for age, gender, race/ethnicity, marriage, education, and smoking status. Model 3 additionally adjusted for hypertension, diabetes, cardiovascular disease, metabolic syndrome, and depression, based on Model 2. We investigated a potential non-linear relationship between 25-hydroxyvitamin D and all-cause mortality in COPD patients using a restricted cubic spline (RCS) to assess continuous 25-hydroxyvitamin D levels, with knots at the 5th, 50th, and 95th percentiles. If non-linear, we performed segmented linear regression for further analysis. We conducted a mediation analysis to assess the potential mediating role of vitamin D between DII and mortality. Lastly, subgroup and sensitivity analyses were performed to confirm the results’ robustness. All regressions have undergone goodness of fit testing. All analyses were performed using R (version 4.2.0).

## Results

### Characteristics of participants

Participant demographics and characteristics are presented in Table 1. Participants were predominantly aged 60 or older (50.75%) and women (60.50%). Additionally, 78.51% identified as non-Hispanic white. Those with higher 25-hydroxyvitamin D levels were more likely to be older, non-Hispanic white, married or cohabitating, highly educated, and non-smokers. They also exhibited lower prevalence of CVD, depression, and metabolic syndrome, with no significant differences in diabetes and hypertension rates.

**Table 1:** Baseline characteristics of participants with COPD according to serum 25(OH)D concentrations.

Characteristics	Serum 25(OH)D Concentrations (nmol/L)				
	Total	<50 (*n* = 592)	50–74.9 (*n* = 615)	> = 75.0 (*n* = 669)	*p-*value
Age, years					**0.004**
40–49	336 (20.83)	127 (25.23)	91 (17.30)	118 (21.99)	
50–59	472 (28.42)	153 (31.04)	155 (24.42)	164 (31.54)	
60–69	618 (31.60)	186 (25.79)	232 (35.41)	200 (31.16)	
70–79	450 (19.15)	126 (17.94)	191 (22.88)	133 (15.31)	
Gender, %					0.09
Female	1,070 (60.50)	344 (62.73)	394 (62.66)	332 (56.04)	
Male	806 (39.50)	248 (37.27)	275 (37.34)	283 (43.96)	
Race/ethnicity, %					**<0.0001**
White	1,088 (78.51)	254 (66.30)	471 (86.11)	363 (78.12)	
Black	378 (8.82)	204 (19.27)	80 (4.27)	94 (6.66)	
Mexican American	126 (2.51)	49 (3.71)	37 (1.98)	40 (2.29)	
Others	284 (10.16)	85 (10.73)	81 (7.65)	118 (12.93)	
Marriage, %					**<0.0001**
Married/Living with partner	959 (58.94)	265 (48.48)	375 (67.27)	319 (56.25)	
Widowed/divorced/separated	740 (33.64)	258 (40.33)	240 (27.30)	242 (36.66)	
Never married	177 (7.42)	69 (11.18)	54 (5.43)	54 (7.09)	
Education, %					
<High school	524 (19.22)	199 (25.67)	140 (13.14)	185 (22.07)	**<0.0001**
High school	492 (28.86)	156 (30.84)	192 (29.40)	144 (26.66)	
>High school	860 (51.92)	237 (43.49)	337 (57.46)	286 (51.26)	
Smoking status, %					**0.002**
Never	496 (27.37)	153 (26.71)	179 (29.43)	164 (25.23)	
Former	687 (35.97)	183 (27.67)	287 (41.22)	217 (35.60)	
Now	693 (36.66)	256 (45.61)	203 (29.35)	234 (39.17)	
DM, %					0.98
Yes	569 (25.54)	189 (25.83)	213 (25.68)	167 (25.13)	
No	1,307 (74.46)	403 (74.17)	456 (74.32)	448 (74.87)	
Hypertension, %					0.15
Yes	1,230 (59.94)	397 (65.37)	446 (57.79)	387 (58.53)	
No	646 (40.06)	195 (34.63)	223 (42.21)	228 (41.47)	
Cardiovascular disease, %					**0.002**
Yes	611 (28.58)	211 (36.92)	203 (23.65)	197 (28.51)	
No	1,265 (71.42)	381 (63.08)	466 (76.35)	418 (71.49)	
Depression, %					**0.001**
Yes	863 (41.31)	295 (50.52)	278 (34.81)	290 (42.60)	
No	1,013 (58.69)	297 (49.48)	391 (65.19)	325 (57.40)	
Metabolic syndrome, %					**0.01**
Yes	1,018 (51.59)	338 (59.32)	363 (48.29)	317 (49.92)	
No	858 (48.41)	254 (40.68)	306 (51.71)	298 (50.08)	
Serum 25(OH)D Concentrations, nmol/L	71.49 (1.44)	35.28 (0.52)	62.95 (0.33)	99.79 (1.60)	**<0.0001**
Dietary Inflammatory Index	1.84 (0.07)	2.27 (0.10)	1.52 (0.09)	1.92 (0.11)	**<0.0001**

Bold values (when *p* is less then 0.05) means it is statistically significant.

### Serum 25(OH)D concentrations and mortality

**Figure 2:**
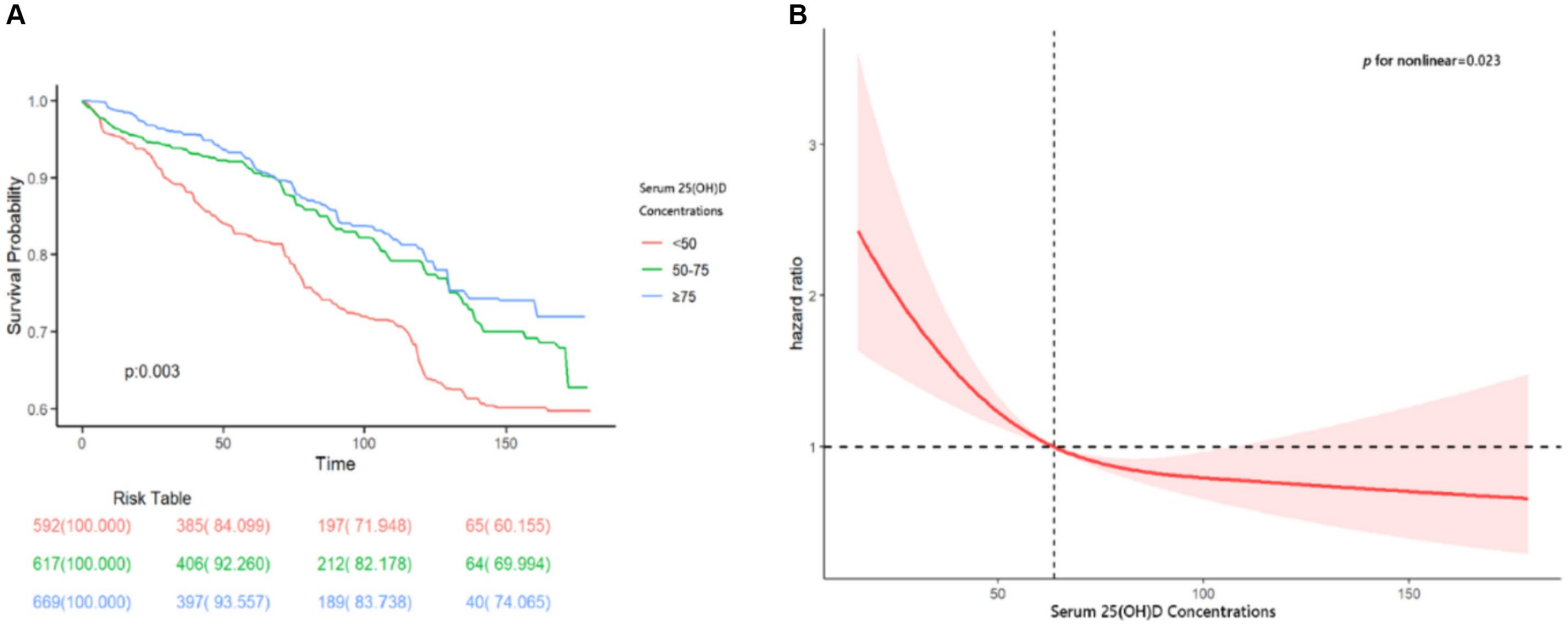
**(A)** The cumulative incidence of all-cause death in the three groups of serum 25(OH)D concentrations during the follow-up period. **(B)** The restricted cubic spline (RCS) analyses between serum 25(OH)D and all-cause mortality of participants with COPD. RCS adjusted for age, gender, race/ethnicity, marriage, education, smoking status, hypertension, diabetes, cardiovascular disease, metabolic syndrome, and depression.

Over the follow-up period, 395 participants died, with a median duration of 70 months. The Kaplan–Meier curve revealed significantly elevated all-cause mortality rates among COPD patients with lower vitamin D levels (*p* = 0.003) (Figure 2A). The Cox proportional hazards model confirmed increased mortality rates for serum 25(OH)D deficiency categories across all models. In the fully adjusted Model 3, the stratified HRs and 95% CIs for serum 25(OH)D categories were as follows: Q1 (<50.0 nmol/L) as reference, Q2 (50.0–74.9 nmol/L) at 0.63 (0.47–0.83), and Q3 (≥75.0 nmol/L) at 0.52 (0.37–0.72), with a significant decreasing trend (*p*-trend <0.001) (Table 2). The RCS analysis demonstrated a non-linear association between serum 25(OH)D levels and all-cause mortality in COPD patients (*p*-nonlinearity = 0.023) (Figure 2B). Additional analyses, including threshold effects and segmented linear regression, were conducted to explore the relationship between serum 25(OH)D levels and all-cause mortality in COPD patients. The findings identified a threshold at 65.3 nmol/L. Above 65.3 nmol/L, serum 25(OH)D levels did not significantly correlate with mortality. Conversely, below 65.3 nmol/L, a negative correlation with mortality was observed across all models (Table 3).

**Table 2:** The relationship between serum 25(OH)D concentrations and all-cause mortality of COPD among participants from the NHANES (2005–2018).

Models	Serum 25(OH)D Concentrations (nmol/L), HR (95% CI)					
	Model 1		Model 2		Model 3	
Character	95% CI	*p*	95% CI	*p*	95% CI	*p*
<50	Ref		Ref		Ref	
50–74.9	0.63 (0.45,0.89)	**0.01**	0.56 (0.43, 0.73)	**<0.0001**	0.63 (0.47, 0.83)	**0.001**
> = 75	0.53 (0.37,0.76)	**<0.001**	0.44 (0.32, 0.62)	**<0.0001**	0.52 (0.37, 0.72)	**<0.001**
*p* for trend		**<0.001**		**<0.0001**		**<0.001**

Model 1: non-adjusted.

Model 2: adjusted for age, gender, race/ethnicity, marriage, education, and smoking status.

Model 3: adjusted for model 2 plus hypertension, diabetes, cardiovascular disease, metabolic syndrome, and depression.

Bold values (when *p* is less then 0.05) means it is statistically significant.

**Table 3:** Threshold effect analysis of serum 25(OH)D concentrations on all-cause mortality in COPD patients.

	Adjusted HR (95% CI), *p*-value
Fitting by the standard linear model	0.99 (0.98, 1.00) < 0.001
Fitting by the two-piecewise linear model	
Inflection point	65.30 nmol/L
25(OH)D concentrations <65.30 nmol/L	0.98 (0.97, 0.99) 0.005
25(OH)D concentrations > = 65.30 nmol/L	0.99 (0.98, 1.00) 0.21

Adjusted for age, gender, race/ethnicity, marriage, education, smoking status, hypertension, diabetes, cardiovascular disease, metabolic syndrome, and depression.

### Causal mediation analysis

In order to discover the potential mediating effect of serum vitamin D on inflammatory diet and mortality in COPD patients, and to provide value for improving prognosis, we conducted a mediation analysis. Firstly, the Cox proportional hazards model shows a positive correlation between DII and all-cause mortality in the COPD population (Supplementary Table S1). Secondly, correlation analysis shows a negative correlation between DII and serum vitamin D levels (Supplementary Table S2). Finally, the mediation analysis results showed a mediation effect of 22% (95% CI, 0.07–0.74) (Table 4), confirming our hypothesis.

**Table 4:** Mediation effects of 25(OH)D on association of DII and all-cause mortality.

Independent variable	Mediator	Total effect		Indirect effect		Direct effect		Proportion mediated, % (95% CI)
		Coefficient (95% CI)	*p* value	Coefficient (95% CI)	*p* value	Coefficient (95% CI)	*p* value	
DII	25(OH)D	−35.48 (−71.12, −6.93)	0.016	−7.55 (−14.63, −2.55)	<0.001	−27.93 (−63.61, −1.23)	0.028	0.22 (0.07, 0.74)

Mediation analysis was adjusted for age, gender, race/ethnicity, marriage, education, smoking status, hypertension, diabetes, cardiovascular disease, metabolic syndrome, and depression.

### Subgroup and sensitivity analysis

**Figure 3:**
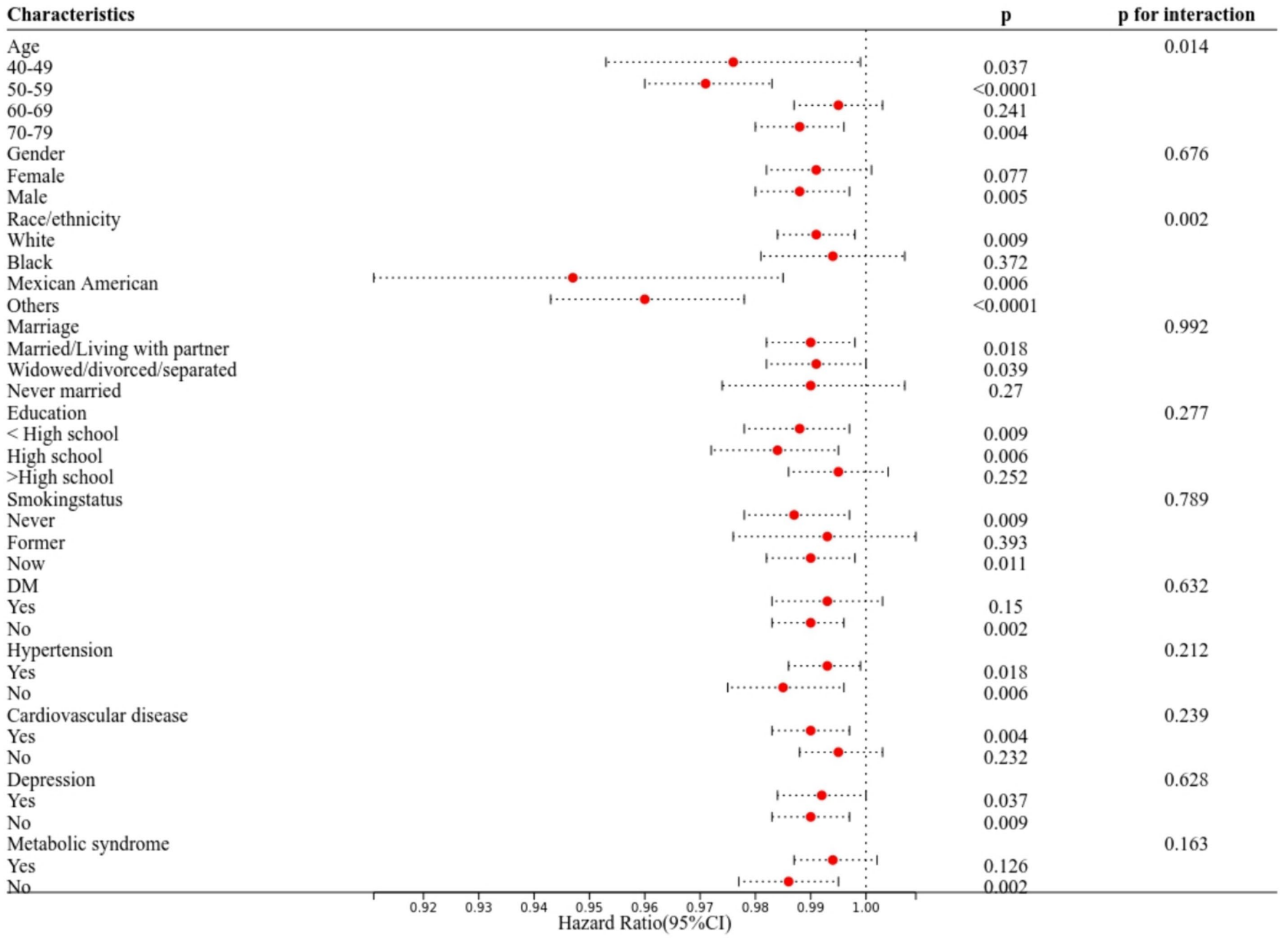
Subgroup analyses of the associations between serum 25(OH)D concentrations and all-cause mortality among participants with COPD from the NHANES (2005–2018). The Cox proportional hazard model was used to estimate the HR of all-cause mortality.

We performed subgroup analyses to assess the influence of demographic factors and comorbidities on the association between serum 25(OH)D concentration and all-cause mortality in patients with COPD. The findings indicated no significant interactions in stratified analyses by sex, marriage, education, smoking status, diabetes, hypertension, cardiovascular disease, metabolic syndrome, and depression (*p* > 0.05). However, significant interactions were observed between serum 25(OH)D concentration and both age and race/ethnicity (Figure 3). Sensitivity analysis, excluding participants with less than 2 years of follow-up and considering 25(OH)D as a continuous variable, produced consistent results (Supplementary Tables S3, S4), confirming the robustness of our findings.

## Discussion

Our study utilized the NHANES mortality cohort data from 2005 to 2018 to assess the relationship between serum 25(OH)D concentrations and all-cause mortality among US COPD patients aged 40 to 79. First, our findings indicate that 64.34% of COPD patients have serum 25(OH)D deficiency, underscoring a widespread prevalence of insufficient vitamin D levels consistent with previous research (26–28). Second, an L-shaped correlation between serum 25(OH)D levels and all-cause mortality was observed in COPD patients, suggesting that, within a specific range, reduced levels significantly associate with increased all-cause mortality. The observed association remains significant, independent of conventional lifestyle factors and prevalent comorbidities such as diabetes, hypertension, cardiovascular disease, and metabolic syndrome, with subgroup analysis corroborating this conclusion. These results may lead to clinical and dietary guidelines appears.

In individuals with COPD, two key factors contributing to breathing difficulties and restricted airflow are inflammation within the bronchial tube linings (obstructive bronchiolitis) and the destruction of alveolar sacs (emphysema) (29). Obstructive bronchiolitis is characterized by mucus cell hyperplasia, an increase in smooth muscle cells, and fibrosis in the airways (30). The development of emphysema is attributed to an imbalance in protease/antiprotease enzyme activity (31). Prolonged chronic inflammation can lead to the generation of endogenous reactive oxygen species (ROS), resulting in an imbalance in oxidants/antioxidants (32). Furthermore, ROS can activate various pro-inflammatory pathways, such as nuclear factor kB (NF k β) and the MAPK pathway, thereby triggering inflammation (33). Studies have indicated a decrease in Nrf2 gene expression in COPD patients, which may contribute to oxidative stress in lung tissue (34). Insufficient levels of vitamin D have been associated with the progression of various pathological processes in COPD. These processes include the regulation of inflammation, heightened oxidative stress, increased expression of proteases, compromised host defense, and remodeling of the pulmonary airway (10). According to a study a deficiency in vitamin D can lead to an increase in the production of several matrix metalloproteinases (MMP2, MMP9, and MMP12), resulting in an imbalance in protease/antiprotease expression (35). Additionally, vitamin D inhibits the TGF-b1 signaling pathway, which is linked to fibrosis in COPD (36). Numerous studies have highlighted the potential antioxidant properties of vitamin D and its analogues, as well as their ability to activate Nrf2 (37). Vitamin D stimulates Nrf2 expression, thereby enhancing the phagocytic potential of alveolar macrophages in COPD patients. Chronic inflammation and oxidative stress play a crucial role in the development of COPD, the related function of vitamin D could potentially serve as an effective therapeutic target.

Several prospective cohort studies suggest an association between vitamin D deficiency and diminished lung function or an increased risk of acute exacerbation in COPD (18, 38, 39). Furthermore, multiple randomized controlled trials (RCTs) have demonstrated that vitamin D supplementation can decrease the incidence of moderate or severe COPD exacerbations in patients with lower baseline concentrations of 25(OH)D (13, 40). Additionally, a meta-analysis incorporating vitamin D and protein gene polymorphism studies has highlighted a connection between vitamin D status and COPD risk (41). Although vitamin D is recognized for its crucial role in bone health and various chronic diseases, the optimal serum 25(OH)D concentration remains contentious. Our findings parallel those of other diseases, specifically the link between low serum 25(OH)D concentrations and all-cause mortality. This relationship is typically non-linear, with mortality rates diminishing as 25(OH)D levels rise up to a threshold point, beyond which no further reduction occurs. Compared with other similar studies, our study has more standardized and high-quality data sources and a longer follow-up time, we also identified a 63.40 nmol/L threshold for all-cause mortality in COPD patients, however, confirming whether serum 25(OH)D concentrations at or above this level mitigate the risk of premature death requires further clinical trials.

Current research suggests that dietary inflammation is associated with the incidence of COPD, deterioration in lung function, and disease progression, potentially linked to the chronic inflammatory nature of such diets contributing to COPD’s progression (42). However, the relationship between diet-related inflammation and COPD mortality remains poorly understood, and it is unclear whether vitamin D deficiency in COPD patients is related to this phenomenon or not. Our study utilized the Dietary Inflammatory Index (DII) to quantify dietary inflammation. DII is designed based on the influence of dietary parameters on inflammatory biomarkers (IL-4, IL-6, IL-10, TNF-α, and CRP), which may stimulate the activation of CYP27B1 (43). CYP27B1 is an enzyme that converts 25 (OH) D into its active form 1,25 (OH)_2_ D. Elevated 1,25 (OH)_2_D can inhibit the conversion of vitamin D_3_ to 25 (OH)D and the liver synthesis of 25(OH)D, consequently resulting in a reduction in serum 25(OH)D levels (44). Findings indicated a positive correlation between DII and COPD mortality, a negative correlation with serum 25(OH)D level. Additional mediation analysis supported serum 25(OH)D’s mediating role between dietary inflammation and mortality. Different from other studies, this new discovery in COPD patients could potentially establish a novel pathway hypothesis, leading to new treatment avenues in the future.

This study has several notable strengths. Initially, the study included a nationally representative sample of American adults with COPD, which ensured the generalizability of the results thanks to the large sample size. Additionally, the extended follow-up period for tracking fatalities provides a robust foundation for the study’s analysis. Secondly, meticulous adjustments for socioeconomic status, dietary and lifestyle factors, comorbidities, and other potential confounders strengthen our conclusions. Finally, using standardized methods to ascertain serum 25(OH)D concentrations in the NHANES database ensures the reliability of our data analysis.

However, this research has its limitations. First, the observational nature of the study does not allow for the establishment of causation. Second, a single 25(OH)D measurement at recruitment may not accurately capture long-term exposure levels. Nonetheless, other studies indicate that a single measurement can adequately reflect vitamin D status over time (45), and a moderate ICC suggests that time-dependent variation is unlikely to significantly affect the study’s findings. Third, the inclusion of COPD patients was based on initial questions without subsequent verification from medical records. Lastly, like other observational studies, this research cannot rule out the possibility of residual or unknown confounding, or unanticipated confounding effects due to measurement errors and unmeasured variables.

In summary, considering multiple factors, this study discovered a significant and consistent association between lower serum 25(OH)D levels and increased risks of death from all causes, as well as its mediating role in the impact of the Dietary Inflammatory Index on mortality among American adults with COPD. This finding could serve as a target for interventions aimed at decreasing the risk of premature death. The findings underscore the importance of monitoring and evaluating vitamin D levels to prevent mortality in adults with COPD and provide a possible preventive approach.

## Data availability statement

Publicly available datasets were analyzed in this study. This data can be found here: NHANES.

## Ethics statement

The studies involving humans were approved by the National Center for Health Statistics. The studies were conducted in accordance with the local legislation and institutional requirements. The participants provided their written informed consent to participate in this study.

## Author contributions

QJ: Conceptualization, Data curation, Methodology, Software, Visualization, Writing – original draft, Writing – review & editing. YJ: Conceptualization, Formal analysis, Supervision, Writing – review & editing. ZM: Conceptualization, Software, Validation, Writing – review & editing. JH: Conceptualization, Methodology, Writing – review & editing. YL: Conceptualization, Investigation, Methodology, Project administration, Resources, Writing – review & editing.
