# Supplementary material for: Nonlinear correlation and mediation effects between serum 25-hydroxyvitamin D levels and all-cause mortality in COPD patients
Source: Front Nutr. 2024 Jun 4;11:1412606. doi: 10.3389/fnut.2024.1412606 (PMC11188383; doi:10.3389/fnut.2024.1412606)
Supplement: Supplementary file 1 [file Table_1.DOCX]

**Supplement table 1.** The relationship between DII and mortality of COPD among participants from the NHANES (2005-2018).

| models | **Dietary Inflammatory Index, HR(95% CI)** | | | | | |
| --- | --- | --- | --- | --- | --- | --- |
|  | Model 1 | | Model 2 | | Model 3 | |
| character | 95%CI | P | 95%CI | P | 95%CI | P |
| DII | 1.08(1.01,1.16) | **0.02** | 1.14(1.06, 1.22) | **<0.001** | 1.10(1.02, 1.18) | **0.01** |

Model 1: non-adjusted

Model 2: adjusted for age, gender, race/ethnicity, marriage, education, smoking status

Model 3: adjusted for model 2 plus hypertension, diabetes, cardiovascular disease, metabolic syndrome, and depression

**Supplement table 2.** Correlation between DII and serum 25(OH)D concentrations among COPD participants from the NHANES (2005-2018).

| 25(0H)D | Model 1 | | Model 2 | | Model 3 | |
| --- | --- | --- | --- | --- | --- | --- |
| character | β(95%CI) | P | β(95%CI) | P | β(95%CI) | P |
| DII | -2.4(-3.53,-1.26) | **<0.0001** | -1.91( -3.15, -0.67) | **0.003** | -1.68( -2.84, -0.52) | **0.01** |

Model 1: non-adjusted

Model 2: adjusted for age, gender, race/ethnicity, marriage, education, smoking status

Model 3: adjusted for model 2 plus hypertension, diabetes, cardiovascular disease, metabolic syndrome, and depression

**Supplement table 3.** HR (95% CIs) for all-cause mortality were determined based on serum 25(OH)D concentrations in COPD participants excluding those with follow-up periods of less than 24 months.

| models | **Serum 25(0H)D Concentrations (nmol/L),HR(95% CI)** | | | | | |
| --- | --- | --- | --- | --- | --- | --- |
|  | Model 1 | | Model 2 | | Model 3 | |
| character | 95%CI | P | 95%CI | P | 95%CI | P |
| <50 | ref |  | ref |  | ref |  |
| 50-74.9 | 0.59(0.39,0.88) | **0.01** | 0.50(0.36, 0.69) | **<0.0001** | 0.56(0.40, 0.78) | **<0.001** |
| >=75 | 0.56(0.35,0.90) | **0.02** | 0.46(0.29, 0.73) | **<0.001** | 0.54(0.34, 0.85) | **0.01** |
| p for trend |  | **0.02** |  | **0.002** |  | **0.01** |

Model 1: non-adjusted

Model 2: adjusted for age, gender, race/ethnicity, marriage, education, smoking status

Model 3: adjusted for model 2 plus hypertension, diabetes, cardiovascular disease, metabolic syndrome, and depression

**Supplement table 4.** HR (95% CIs) for all-cause mortality were determined based on serum 25(OH)D concentrations(continuous) in COPD participants

| models | **Serum 25(0H)D Concentrations (nmol/L),HR(95% CI)** | | | | | |
| --- | --- | --- | --- | --- | --- | --- |
|  | crude model | | Model 1 | | Model 2 | |
| character | 95%CI | P | 95%CI | P | 95%CI | P |
| 25(OH)D | 0.99(0.98,1.00) | **0.001** | 0.99(0.98, 0.99) | **<0.0001** | 0.99(0.98, 1.00) | **<0.001** |

Model 1: non-adjusted

Model 2: adjusted for age, gender, race/ethnicity, marriage, education, smoking status

Model 3: adjusted for model 2 plus hypertension, diabetes, cardiovascular disease, metabolic syndrome, and depression
